# Deconvolution of diffuse gastric cancer and the suppression of CD34 on the BALB/c nude mice model

**DOI:** 10.1186/s12885-020-06814-4

**Published:** 2020-04-15

**Authors:** Seon-Jin Yoon, Jungmin Park, Youngmin Shin, Yuna Choi, Sahng Wook Park, Seok-Gu Kang, Hye Young Son, Yong-Min Huh

**Affiliations:** 1 Department of Biochemistry and Molecular Biology, Yonsei University College of Medicine, Seoul, South Korea; 2 Brain Korea 21 PLUS Project for Medical Science, Yonsei University, Seoul, South Korea; 3 Department of Radiology, Severance Hospital, Yonsei University College of Medicine, Seoul, Republic of Korea; 4 Departments of Neurosurgery, Severance Hospital, Yonsei University College of Medicine, Seoul, South Korea; 5 Department of Medical Science, Yonsei University Graduate School, Seoul, South Korea; 6 Severance Biomedical Science Institute, College of Medicine, Yonsei University, Seoul, South Korea; 7 YUHS-KRIBB Medical Convergence Research Institute, Seoul, South Korea

**Keywords:** Diffuse gastric cancer, CD34, Knockdown, Magnetic resonance imaging, Histology, Phenotype, BALB/c nude mouse

## Abstract

**Background:**

Gastric cancer is a considerable burden for worldwide patients. And diffuse gastric cancer is the most insidious subgroup with poor survival. The phenotypic characterization of the diffuse gastric cancer cell line can be useful for gastric cancer researchers. In this article, we aimed to characterize the diffuse gastric cancer cells with MRI and transcriptomic data. We hypothesized that gene expression pattern is associated with the phenotype of the cells and that the heterogeneous enhancement pattern and the high tumorigenicity of SNU484 can be modulated by the perturbation of the highly expressed gene.

**Methods:**

We evaluated the 9.4 T magnetic resonance imaging and transcriptomic data of the orthotopic mice models from diffuse gastric cancer cells such as SNU484, Hs746T, SNU668, and KATO III. We included MKN74 as an intestinal cancer control cell. After comprehensive analysis integrating MRI and transcriptomic data, we selected CD34 and validated the effect by shRNA in the BALB/c nude mice models.

**Results:**

SNU484, SNU668, Hs746T, and MKN74 formed orthotopic tumors by the 5 weeks after cell injection. The diffuse phenotype was found in the SNU484 and Hs746T. SNU484 was the only tumor showing the heterogeneous enhancement pattern on T2 images with a high level of CD34 expression. Knockdown of CD34 decreased the round-void shape in the H&E staining (*P* = 0.028), the heterogeneous T2 enhancement, and orthotopic tumorigenicity (100% vs 66.7%). The RNAseq showed that the suppressed CD34 is associated with the downregulated gene-sets of the extracellular matrix remodeling.

**Conclusion:**

Suppression of CD34 in the human-originated gastric cancer cell suggests that it is important for the round-void histologic shape, heterogeneous enhancement pattern on MRI, and the growth of gastric cancer cell line.

## Background

Gastric cancer (GCA) is the 5th most common neoplasm worldwide. And the incidence of GCA is profound in South Korea, Mongolia, China, and Japan [1]. Even though early detection of the tumor can prolong the survival of patients [2, 3], about 33% of patients with gastric cancer (GCA) are diagnosed with diffuse GCA (DGCA) [4]. As the name implies, DGCA infiltrates the contour of the stomach insidiously, which lead to the incomplete resections. Some portions of DGCA patients show a phenotype of signet-ring cancer (SRC) [5], and the clinical significance of the signet-ring shape is controversial [6–8].

Even with the importance of DGCA, its biological function is difficult to analyze in the animal orthotopic models, which requires a microsurgical technique [9–11]. Furthermore, the characterization of the shape and the extent of stomach cancer is one more difficulty. When the malicious phenotypes of DGCA can be associated with the transcriptomic data, more opportunities can be obtained in the research of stomach cancer.

To resolve these unmet needs, we hypothesized that a phenotype of DGCA is associated with the cell-specific gene expression and that such phenotype can be suppressed by the knockdown of the gene and thereby showing a tumor-suppressive effect in the BALB/c animal model of gastric cancer.

## Methods

### Cell culture

Human GCCs such as SNU484 (KCLB Cat# 00484, RRID:CVCL_0100), SNU668 (KCLB Cat# 00668, RRID:CVCL_5081), MKN74 (KCLB Cat# 80104, RRID:CVCL_2791), Kato III (KCLB Cat# 30103, RRID:CVCL_0371), and Hs746T ((KCB Cat# KCB 2013048YJ, RRID:CVCL_0333) were purchased from the Korean Cell Line Bank (Seoul, Korea). SNU-484, SNU-668, MKN-74, and Kato III were grown in RPMI 1640 (Welgene, Daegu, Korea); Hs746T was grown in Dulbecco’s modified Eagle’s medium (DMEM, Welgene); All cells were cultured with 1% antibiotic-antimycotic solution (including 10,000 units penicillin, 10 mg streptomycin, and 25 μg amphotericin B per mL, Sigma-Aldrich) at 37 °C in a humidified atmosphere containing 5% CO_2_. All cells were confirmed to be negative for mycoplasma by e-Myco™ plus Mycoplasma PCR Detection Kit (iNtRON Biotechnology, Seongnam, Korea). Cell lines were supplemented with 10% FBS (Gibco) as the basis for the analysis. Four replicates of each cell were introduced for microarray data. These cell lines were used in the animal experiments.

### Invasion assay

2 × 10^4^ HUVEC cells in a culture medium (M199) were added to a transwell coated with fibronectin. The bottom of the well was coated with 0.2% gelatin and subsequently incubated for 48 h until the formation of a monolayer. Then, 1 × 10^5^/50 μL GCCs (SNU484, SNU668, Hs746T, and other cells such as YCC1, YCC2, YCC3, YCC6, YCC16, MKN28, AGS) with CellTracker™ (Molecular Probes, C2925) without FBS were separately added to the transwell. Culture medium with 10% FBS was added to the lower chamber. After incubation for 48 h, the upper cells of the membrane were removed with a cotton swab. Cells on the lower membrane were lysed with 200 μL lysis buffer for 2–3 h at room temperature. Fluorescence was measured with Ex/Em 492/517.

### Animal models

All animal experiments were conducted with the approval of the Institutional Animal Care and Use Committee of the Yonsei Laboratory Animal center (IACUC 2017–0329). Four-week-old male BALB/c nude mice (*N* = 60, average weight 22 ± 2 g) were purchased from Orient Bio (Seongnam, South Korea). The sample size of animal was based on the prior reports [12, 13]. After 1 week of acclimatization, each group of five mice is housed in the separated individual standard cleaned cages under automatically controlled air condition system with temperature (22 ± 2 °C), humidity (about 60%), and lighting (12:12-h light–dark cycle). Diet and sterilized water are provided ad libitum throughout the experiments. Mice were modeled simultaneously for orthotopic and heterotopic site after randomization of the size of the mice. After all images were taken for the specified models, the mice were euthanized with CO_2_, and the collected stomach samples were sent for H&E staining.

### Orthotopic mice model

Orthotopic modeling required operation with anesthesia (Isoflurane 2%) [14]. Cells were injected (1 × 10^7^ GCCs/30 μl PBS, 29G insulin syringe) into the walls of the exteriorized stomachs of BALB/c nude mice (male) by incising the skin and peritoneum along the upper midline for approximately 5 mm following the method. The stomach was returned to the peritoneum, and the abdominal wall was closed with a wound suture in one layer.

### Heterotopic xenograft mice model

1 × 10^7^ GCCs were transplanted into the proximal thigh region of BALB/c nude mice (male), and we observed the model until the tumor volume expanded up to 4000 mm^3^. Implanted tumor size was assessed three times per week and calculated by (4/3) × π × (minor axis/2)^2^ × (major axis/2) mm^3^. Tumorigenicity was measured in the third week after injecting the cells (*n* = 10 per group). We plotted the KM plot not by the survival of the mice but by the 4-fold size change from the initial measurement as the event. If the diagonal tumor size was larger than 2 cm, the mice were euthanized with CO_2_.

### Animal magnetic resonance imaging

Mice were imaged on a 9.4 T animal MRI with a Bruker animal coil (RF SUC 400 1H M-BR-LIN ROAD, Bruker Medical Systems, Massachusetts, USA) after anesthesia with isoflurane (2% maintained throughout the study with nose cone). Each mouse was kept at approximately 37 °C while in the scanner. Mice were scanned during the day time (light cycle) two to four times during the orthotopic model imaging depending on the established cell types (SNU484, First image 2 weeks after injection, three times with a week interval; SNU668 first image 1 week after injection, four times images with 1 to 2-week interval; Hs746T image was taken once 19 days after injection; MKN74 first image 17 days after injection, four times with a week interval; Kato III first image 4 weeks after injection, two times with 1-week interval). Sequences were adopted at room temperature with the following parameters: Echo = 1, TR = 2300 ms, TE = 22.0 ms, FA = 180 deg, TA = 0 h 4 m 54 s 400 ms, NEX = 2, and FOV = 4.00 cm. After taking the MR image, mice were sent back to the same cleaned cage.

### Knockdown of CD34

Lentiviral particles (CD34 shRNA particle; sc-29,249 V, Control shRNA particle; sc-108,080, Santa Cruz Biotechnology, USA) for CD34 suppression were purchased from Santa Cruz. There are three groups in this experiment: wildtype (SNU484 WT), scramble shRNA control (SNU484 SC), and CD34 knockdown cells (SNU484 KD). SNU484 WT cell line was plated on the 12-well plate 24 h prior to the infection for 50% confluence on day 2. Polybrene pretest was maintained far below 5 μg/ml. Lentiviral shRNA was transfected on day two following the manufacturer’s instructions. Puromycin-resistant clones were subcultured and expanded, and CD34 expression was assessed. Mice models were established using SNU484 SC and SNU484 KD (Five mice for each group, Same setting with a replication, Total twenty mice).

### Tumorsphere formation assay

We compared the tumorsphere formation between SNU484 SC and SNU484 KD. In 96-well plates, ten cells of each type were cultured in 50 μL DMEM/F12 (Gibco) supplemented with bFGF, EGF, B27, 10% FBS, and 1% antibiotics. After 30 days of incubation, spheres in each well were counted for statistical comparison.

### Migration assay

Three types of SNU484 cells were grown in monolayers in culture media, with 10% FBS and 1% antibiotics. When the confluency reached 70%, cell monolayers were scratched with a 100 μL pipette tip. Wound width was measured after 72 h and then normalized by wound width measured immediately after scratching.

### Drug sensitivity agents

Three types of cells were cultured for a drug sensitivity experiment: SNU484 WT, S484 SC, and S484 KD with RPMI media as a negative control. S484 WT (1 × 10^4^ cells/well) cells in 96-well plates were treated with 100 μl of 5- Fluorouracil (5-FU, Sigma, Cat. F6627-5G) at a concentration of 155 μM with RPMI 1640 and without FBS. 5-FU was dissolved in DMSO. Oxaliplatin 100 μl was administered at a concentration of 5 μM. The combination of the drug is 100 μl of each drug (0.07 μl of 5-FU, 0.04 μl of oxaliplatin). Drug sensitivity was measured with CCK at 24 h, 48 h, and 72 h after treatment with the drugs.

### H&E staining

Histology sections were compared among the SNU484 SC, SNU484 KD, MKN74, and SNU668 and they were magnified (100 X) using the image software Olyvia 2.5 (Olympus) with the image field size of 1186 px by 668 px for a single analysis. H&E staining of the samples was thoroughly examined. SNU484 SC, and SNU484 KD were compared to assess the growth patterns and signet-ring phenotypes.

### Human microarray

The microarray (Illumina HT12 v3, United States) of GCA patients of Severance Hospital (Republic of Korea, Seoul, from 2000 to 2010, IRB 4–2016-0013) was analyzed for the effects and the associations of molecular markers [15]. A pathologist and surgeon both confirmed the Lauren classification and tumor size. TNM staging was designated according to the AJCC 8th edition. Histology was divided into Moderately-differentiated (MD), Poorly-differentiated (PD), Signet-ring carcinoma (SRC), Well-differentiated (WD).

### RNA sequencing

We collected samples of SNU484 WT, SNU484 SC, and SNU484 KD and compared differential gene expression among these samples. The sequencing platform is Hiseq 2500 with total RNA prepped with Truseq stranded total RNA H/M/R prep kit (Illumina, United States). Diffuse gastric cancer cells, SNU484 and Hs746T were revalidated with an RNAseq (Novaseq6000) prepared with the TruseqStranded mRNA Prep Kit. Tophat (version 2.0.13) was used to map the FASTA on the hg19 human reference chromosome for both RNAseq analysis. FPKM data was obtained using cufflink (Version 2.2.0) with a mapping rate of more than 87%.

### Data analysis

By using the expression level of CD34 in the tumor patients, we summarized the clinical variables. We compared the Cox hazard ratio for CD34, which is adjusted for age, sex, Charlson comorbidity score (CCI), tumor size, location, Lauren classification, histologic type, and AJCC TNM stage (8th edition) according to each selected subgroup. Two-sided Student t-test was used for the analysis. For gene extraction, we used the Elastic-net algorithm and t-test [16].

### Data availability

All the patient microarray data analyzed in this study are open to the public and can be downloaded from GEO (Yonsei gastric cancer cohort, YGC, GSE84433; Cell line microarray GSE146361).

## Result

### The tumorigenic potential of cell lines

**Fig. 1:**
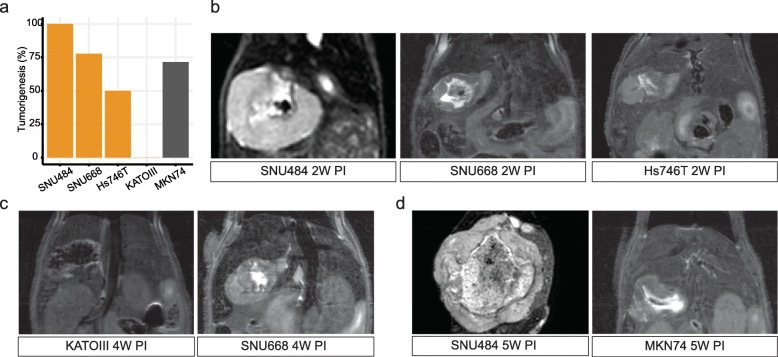
Tumorigenesis in the BALB/c mouse. **a** The overall tumorigenesis rate of orthotopic models. **b** 9.4 T MRI demonstrates the orthotopic tumor 2 weeks after the injection. **c** The orthotopic models 4 weeks after the injection. **d** Growing size of tumors were measured with the MRI. The same number of cells (1 × 10^7^ cells) were injected to the stomach surface of BALB/c mice**. MRI:** Magnetic resonance imaging, **PI:** Post-injection

Our research begins with a hypothesis that diffuse gastric cancer cells (DGCA GCCs) will form a diffuse infiltrating tumor in the mice model. We selected DGCA GCCs from commercially available sources. DGCA GCCs such as SNU484, Hs746T, SNU668, and KATO III were included for our study. Intestinal type GCC MKN74 was used as a control. We evaluated the tumorigenic potential in the orthotopic BALB/c nude mice model. All of the five cell lines could establish the tumor in the heterotopic mice model at 2 weeks after injection of cells more than 90% of the trials (Table S1). We established an orthotopic mice model that exteriorized stomachs after excision of skin and peritoneum of the mice under anesthesia. The same number of cells were injected (1 × 10^7^ GCCs/30 μl PBS), and the tumorigenic potential decreased from the heterotopic model (Fig. 1a, Table S1).

The robust tumorigenic cell was SNU484 (100%, 8/8 mice) followed by SNU668 (77.7%, 7/9), Hs746T (50%, 5/10 mice), and MKN74(71.4%, 5/7 mice). Kato III not established any tumor in the orthotopic models while showing 100% tumorigenicity in the heterotopic models (Table S1). We assessed the radiologic pattern of orthotopic mice models with 9.4 T MRI T2 images (Fig. 1). SNU484 (75%, 6/8 mice) and Hs746T (40%, 2/5 mice) were the diffuse phenotype cells while SNU668 (14.3%, 1/7 mice) and MKN74 (0%, 0/5 mice) showed less than 50% of diffuse shape in the MRI up to 5 weeks of follow up (Fig. 4a). Most distinctively, SNU484 showed a heterogeneous enhancement pattern in the T2 image without contrast agent (Fig. 1).

### Deconvolution of gastric cancer cells

**Fig. 2:**
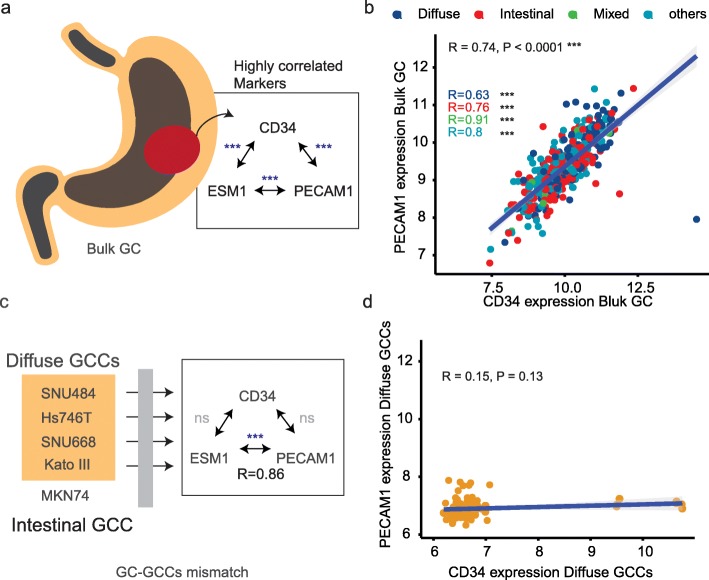
Different expression patterns of three genes of gastric cancer. **a** High correlations among the three marker genes. **b** The microarray result of YGC cohort. **c** Loss of the correlations among the three genes. **d** the result of Diffuse GCCs. **GC:** gastric cancer, **GCCs:** gastric cancer cells, **ns:** statistically not significant

We began with a hypothesis that diffuse gastric cancer cell lines are molecularly different from other cell lines. We used the elastic-net algorithm and student t-test to find the cell-specific markers of DGCA GCCs (Fig. 2c, Fig. S11) [16]. We found CD34, ESM 1, and PECAM1 are differentially expressed in the SNU484 and Hs746T cell lines (Fig. S1).

We explored the bulk tissue data and cell microarray data, and we found a disparity between these two databases. CD34 gene has been suspected as the gastric cancer cell marker, and it is associated with the microvascular density and poorly differentiated histological type in the human [17, 18]. In the bulk gastric cancer tissue microarray data, CD34 shows a high correlation with other vascular markers, ESM 1 and PECAM1 (Fig. 2a, b) [19–21]. The three pairs from the three genes show statistically positive results (*P* < 0.0001, *N* = 357). Histologic subtypes also showed statistical significance (*P* < 0.0001) between CD34 and PECAM1. Two other associations preserved significant results in the diffuse, intestinal, other histologic types (*P* < 0.0001), and in the mixed type (*P* < 0.01).

We estimated the Cox hazard ratio of CD34 level of bulk GC samples, and it is associated with the survival of GC patients (Table 1). Such association may be contributed by the higher proportion of diffuse Lauren type or the PD or SRC histologic type of GC in the subpopulation (Table S2). We included different covariates to the models, and the associations of CD34 and the survival were robust in the models (Table S3). We also did subgroup analysis and found CD34 level is more dominantly associated in the older age, poorly differentiated histology, and the AJCC grade more than 3B (Table S4). And such clinical characteristics lead us to hypothesize that CD34 and these related markers (ESM 1 and PECAM1) might be involved with a more malicious phenotype of GC.

**Table 1:** Multivariate Cox hazard model of YGC cohort with tissue expression of CD34 (*N* = 357)

VARIABLES		N	HR (95% CI)	*P* value
Age				
27–61		180	Reference	
62–86		177	1.703 (1.244–2.331)	< 0.001
Sex				
Female		115	Reference	
Male		242	1.195 (0.856–1.668)	0.296
Histology				
MD		110	Reference	
Other		75	1.248 (0.79–1.973)	0.342
PD		172	1.411 (0.981–2.029)	0.064
TNM 8th				
I to IIB		112	Reference	
IIIA		112	2.821 (1.741–4.573)	< 0.001
IIIB to IV		133	5.142 (3.319–7.967)	< 0.001
Chemotherapy				
Yes		294	Reference	
Other		63	2.593 (1.702–3.95)	< 0.001
Microarray				
CD34 Expression		357	1.241 (1.046–1.473)	0.014

*MD* moderately differentiated, *PD* poorly differentiated, *TNM* the classification of malignant tumors, *HR* hazard ratio, *CI* confidence interval

We assessed the cell microarray, CD34 expression pattern of GCCs is decoupled from the tissue data (Fig. 2c, d). CD34 and the other genes no longer show any correlation except ESM 1 and PECAM1 in the DGCA GCCs (Fig. 2c, *P* < 0.0001, *R* = 0.86) and all GCCs (Fig. S2, *P* < 0.0001, *R* = 0.61).

### The CD34 knockdown decreased tumorigenicity but not altered the diffuse phenotype

**Fig. 3:**
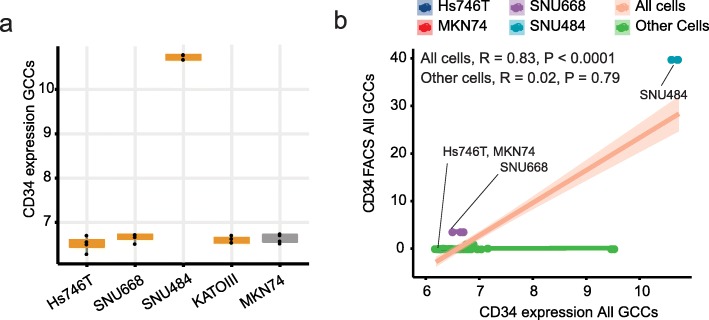
CD34 expression in the diffuse gastric cancer cell lines. **a** Microarray gene expression. **b** CD34 microarray correlated with the CD34 antibody. **GCCs:** Gastric cancer cell lines

As SNU484 robustly establishes the tumor in the orthotopic model than Hs746T (100% vs. 50%) with the heterogeneous enhancement on T2 MRI (Fig. 1), we focused on SNU484 cells which expressed CD34 gene higher than other cell lines (Fig. 3a). The elevated CD34 mRNA levels of SNU484 were replicated in the protein level assays (Fig. 3b, Fig. S3, Fig. S4).

We used shRNA viral particles to suppress the level of CD34 in the SNU484 cell line. Western blot and flow cytometry showed a decreased expression of CD34 protein level in the SNU484 CD34 KD (Knock-down) than the CD34 SC (Scramble) and CD34 WT (Wildtype) (Fig. S3, S4). SNU484 CD34 KD decreased tumorigenicity from 100 to 66.7% (Table S1), which is compatible with the decreased tumorsphere forming potential than the CD34 SC (Fig. S5, *P* = 0.014). Migration capacity decreased in the SNU484 CD34 KD than the SNU484 SC and SNU484 WT (Fig. S6). However, when injected into the BALB/c nude mice, the diffuse infiltrating pattern of SNU484 remained in the multiple samples in the SNU484 SC and SNU484 CD34 KD (Fig. S7) 40 days after the orthotopic model establishment.

Unlike the gross infiltrating pattern in the MRI, the size of the SNU484 CD34 KD slightly decreased from the SNU484 SC in the heterotopic model (Fig. S8). The survival curve that estimated the event as the point of four-fold increase from the baseline of the heterotopic tumor size showed no statistical significance (Fig. S9, *P* = 0.051).

### The round void shape decreased significantly after CD34 knockdown

**Fig. 4:**
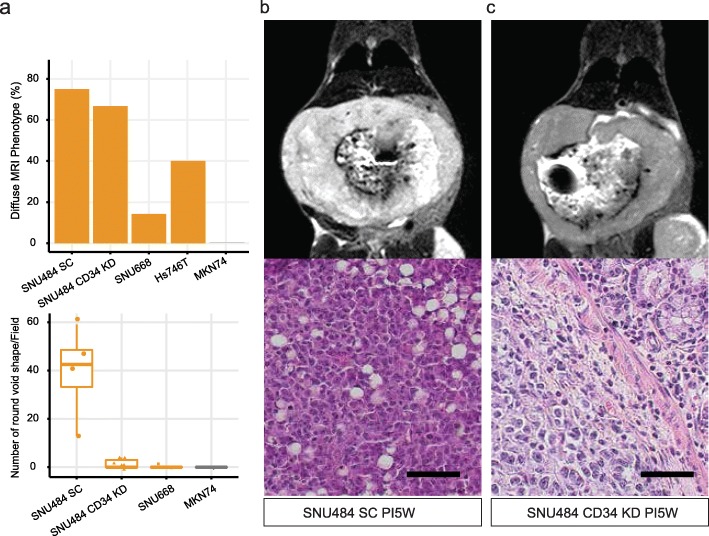
Histological difference after the suppression of CD34. **a** Quantification of diffuse MRI phenotype (Diffuse mice/all injected mice) and the number of round void shape in the H&E staining. **b** SNU484 SC injected mouse MRI and H&E staining. **c** SNU484 CD34 KD MRI and H&E staining. **PI5W:** Five weeks after injection of the cells. **SC:** scramble shRNA treated sample, **KD:** CD34 knockdown. Black bar indicates 50 μm with 100 x magnification

A notable difference was found in the microscopic evaluation of the SNU484 samples from other GCCs (Fig. 4a lower panel). The SNU484 tumors were collected 40 days after the establishment of the models. SNU484 showed a round void shape (RVS) through the slides, which resembles the signet-ring shape of clinical samples (Fig. 4b). SNU484 CD34 KD showed no such area with the RVS (Fig. 4c). We counted the H&E slides and summarized the result in the lower panel of Fig. 4a. SNU484 SC showed a mean of 39 RVS in a single field (Standard deviation [SD] = 19.2 RVS/field). While SNU484KD shows average of 1.3 RVS (SD = 2.0 RVS/field) with a statistical difference from SNU484 SC (*P* = 0.028, *N* = 10). Other GCCs such as SNU668 and MKN74 showed no signet-ring like shape in the formalin-fixed slide of the in vivo tumor.

**Fig. 5:**
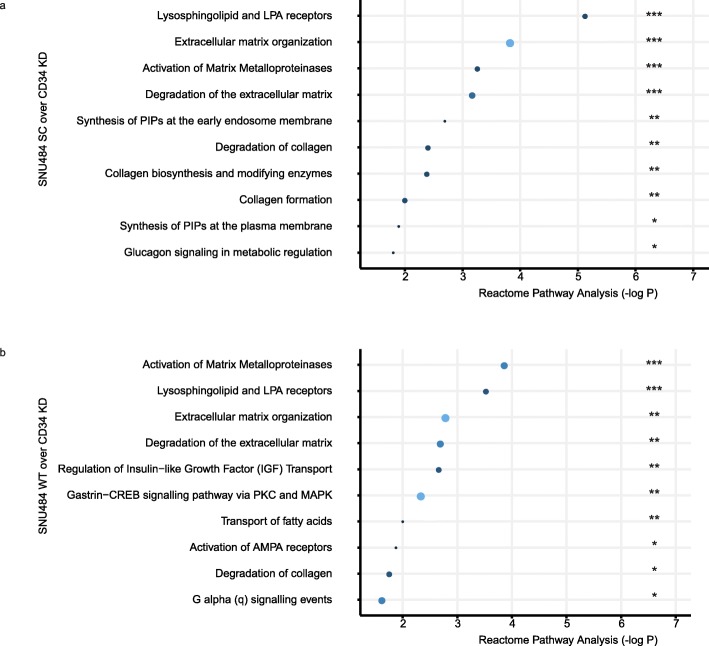
The down-regulated signatures after the CD34 suppression. RNAseq-based differential expression of genes downregulated in the SNU484 CD34 KD cells. The larger size and the light color indicate the number of hit genes. Three stars *P* < 0.0001, Two stars *P* < 0.01, One star *P* < 0.05

We assessed the gene expression pattern of SNU484 CD34KD genes by comparing it with the SNU484 WT and SNU484 SC (Fig. 5). We assessed the downregulated genes with the fold change more than two. In the Reactome database [22], there are at least five top pathways overlapped between the two results. Lysosphingolipid-related pathways (S1PR1, LPAR2, and S1PR5) and extracellular matrix-related pathways (COL4A5, MMP1, MMP10, PLOD2, TIMP1, and COL21A1) were the dominantly downregulated in the CD34 KD SNU484 cells (Fig. 5). We included the details of genes in each pathway in Table S5. However, such perturbation on CD34 not change the drug responsibility against 5-FU or oxaliplatin which are effective in human gastric cancer patients (Fig. S10) [23].

## Discussion

CD34 has been associated with the tissue vascularity and poorly differentiated histologic aspects of human GC [18]. However, the biological function of the gene has not been evaluated in the GC. In this study, we hypothesized that the knockdown of CD34 lead to a phenotypic difference of the SNU484, a GCC with a high level of CD34 expression. Our study found that CD34 might be associated with the signet-ring shaped histology of GC, which disappeared after the knockdown of CD34 (Fig. 4).

Tissue vascularity of CD34 had been in the overlapping domain of tissue stem cells and the hematopoietic stem cells [17, 24]. We assessed tissue microarray data, and it shows high correlations among the levels of CD34 expression and other vessel-related markers (Fig. 2a). However, our study revealed that these markers are decoupled in GCCs (Fig. 2c), and we compared two DGCA GCCs, Hs746T and SNU484. Non-invasive imaging with 9.4 T MRI showed a robust pattern of the tumorigenic potential of SNU484 from Hs746T (Fig. 1). We focused on the SNU484 with a high level of CD34. MRI T2 images showed a heterogeneous pattern of enhancement, a signal of compartmentalized fluid, which disappeared after the knockdown of CD34 in the SNU484 cells (Figs. 4, [25, 26]). Tissue H&E staining revealed there is no definite vascular structure and red blood cells in the in vivo tumor (Fig. 4). With these findings, the void shape is likely to be filled with fluid, and which has not been assessed in this study. Such a phenomenon might come from the downregulation of genes involved in the degradation process of the extracellular matrix (Table S5).

The diffuse phenotype of GCCs was evaluated with MRI in the orthotopic mice models (Fig. 4). SNU484 and Hs746T were likely to show a diffuse phenotype in the orthotopic models. We found that the downregulation of CD34 is not associated with the diffuse phenotype of SNU484 (Fig. 4). However, the high tumorigenic potential of SNU484 cell line in the orthotopic mice was decreased by the CD34 knockdown from 100 to 66.7% (Table S4). SNU668 and Hs746T show about 50 to 70% of tumorigenic potential, which remained until the end of the orthotopic model experiments. In contrast, KATO III initially formed a mass in the heterotopic model and disappeared after 2 weeks. The orthotopic model of KATO III was not established in our BALB/c mice cohort (Table S4).

The genes downregulated from the knockdown of CD34 may implicate a gene set associated with the signet-ring shape of DGCA. The Signet-ring shape is found in the digestive tract cancers, such as esophageal cancer, gastric cancer, and colon cancer [27–30]. And its prognostic effect is still debatable [6, 7, 28]. And the biological component associated with the ring shape was mucin [31, 32]. In this study, we propose the extracellular matrix related genes and lysosphingolipid-related genes (or sphingosine-1-phosphate related genes) as the associated genes with CD34 and the signet-ring phenotype [33, 34]. And recently, there are reports on the cancer progression with lysosphingolipid-related pathway [35]. We speculate that targeting such vulnerabilities would be an option for clinical researchers.

The limitation of our study is associated with the selection of the animal model and the small number of samples. We conducted our study according to the ARRIVE guideline to increase the transparency of our study and minimize unnecessary animal research [36]. SNU484 was the only CD34 high expressing GCC in our database, and it limited the extent of our study and the generalizability of our results. Magnetic resonance imaging was taken at least twice for each animal for the non-invasive evaluation, and the date was harmonized in the same tumor cell group. However, the date of MRI evaluation was not exactly harmonized among other types of GCCs (Fig. 1). We were not able to see the natural survival of animals, and we euthanized the animals when the weight of mice decreases by more than 20% from the baseline (Fig. S8). The survival curves were calculated with the tumor volume as the surrogate marker without definite statistical significance (Fig. S9, *P* = 0.051). The CD34 knockdown was not associated with the different response against 5-FU or oxaliplatin in this study (Fig. S10). And we omitted the administration dose-response relationship in our study.

## Conclusions

We evaluated the knockdown effect of CD34 in the human gastric cancer cell, SNU484, within the BALB/c nude mice models. The most dominant effect was the loss of round-void shape in the H&E staining of the extracted tumor. Such phenotype may come from the genes involved in the remodeling of the extracellular matrix.

## Supplementary information

**Additional file 1 Figure S1.** Three vessel markers of GCCs. **Figure S2.** Correlation of ESM 1 and PECAM1 in the GCCs. **Figure S3.** CD34 expression with antibody from SNU484 GCC. **Figure S4.** Flow cytometry results of SNU484. **Figure S5.** Tumorsphere formation in the SNU484 GCCs. **Figure S6** Migration assay in the SNU484 GCCs. **Figure S7.** Individual comparison of MR images of control and CD34 KD mice. **Figure S8.** The tumor volume in the heterotopic models. **Figure S9.** The KM curve with the survival of orthotopic models. **Figure S10.** Drug treatment on the SNU484. **Figure S11.** RNAseq validation of the SNU484 and Hs746T. **Table S1.** Characteristics of selected GC cell lines. **Table S2.** Clinical information of GC enrolled in this study according to CD34 expression. **Table S3.** Statistical models of CD34 level in the YGC cohort (*N* = 357). **Table S4.** Subgroup analysis of YGC cohort by the tissue level of CD34. **Table S5.** Downregulated genes in the SNU484 CD34 KD.

## Data Availability

All the patient microarray data analyzed in this study are open to the public and can be downloaded from GEO (Yonsei gastric cancer cohort, YGC, GSE84433; Cell line microarray, GSE146361).

## Abbreviations

AJCC: American Joint Committee on Cancer
DEG: Differential expression of genes
DGCA: Diffuse gastric cancer
EMT: Epithelia-Mesenchymal Transition
FPKM: Fragments Per Kilobase of transcript per Million
GCCs: Gastric cancer cells
Hs746T: Name of a cell line used in this research
Kato III: Name of a cell line used in this research
KD: CD34 Knockdown with shRNA particle
MKN74: Name of a cell line used in this research
MRI: Magnetic resonance imaging
PIW: Post-injection week
RPPA: Reverse phase protein lysate microarray
SC: scramble shRNA treated sample
SNU484: Name of a cell line used in this research
SNU668: Name of a cell line used in this research
WT: Wildtype (untreated for CD34 or control shRNA)
YGC: Yonsei gastric cancer cohort
