# Supplementary material for: Deconvolution of diffuse gastric cancer and the suppression of CD34 on the BALB/c nude mice model
Source: BMC Cancer. 2020 Apr 15;20:314. doi: 10.1186/s12885-020-06814-4 (PMC7160933; doi:10.1186/s12885-020-06814-4)
Supplement: Supplementary file 1 — Additional file 1 Figure S1. Three vessel markers of GCCs. Figure S2. Correlation of ESM 1 and PECAM1 in the GCCs. Figure S3. CD34 expression with antibody from SNU484 GCC. Figure S4. Flow cytometry results of SNU484. Figure S5. Tumorsphere formation in the SNU484 GCCs. Figure S6 Migration assay in the SNU484 GCCs. Figure S7. Individual comparison of MR images of control and CD34 KD mice. Figure S8. The tumor volume in the heterotopic models. Figure S9. The KM curve with the survival of orthotopic models. Figure S10. Drug treatment on the SNU484. Figure S11. RNAseq validation of the SNU484 and Hs746T. Table S1. Characteristics of selected GC cell lines. Table S2. Clinical information of GC enrolled in this study according to CD34 expression. Table S3. Statistical models of CD34 level in the YGC cohort (N = 357). Table S4. Subgroup analysis of YGC cohort by the tissue level of CD34. Table S5. Downregulated genes in the SNU484 CD34 KD. [file 12885_2020_6814_MOESM1_ESM.docx]

Deconvolution of diffuse gastric cancer and the suppression of CD34 on the BALB/c nude mice model.

Seon-Jin Yoon, Jungmin Park, Youngmin Shin, Yuna Choi, Sahng Wook Park, Seok-Gu Kang, Hyeyoung Son, and Yong-Min Huh

**Supplementary Material**

| Description |
| --- |
| Appendix Figures |
| Fig. S1 Three vessel markers of GCCs. |
| Fig. S2 Correlation of ESM1 and PECAM1 in the GCCs. |
| Fig. S3 CD34 expression with antibody from SNU484 GCC. |
| Fig. S4 Flow cytometry results of SNU484. |
| Fig. S5 Tumorsphere formation in the SNU484 GCCs. |
| Fig. S6 Migration assay in the SNU484 GCCs. |
| Fig. S7 Individual comparison of MR images of control and CD34 KD mice. |
| Fig. S8 The tumor volume in the heterotopic models. |
| Fig. S9 The KM curve with the survival of orthotopic models. |
| Fig. S10. Drug treatment on the SNU484. |
| Fig. S11. RNAseq validation of the SNU484 and Hs746T. |
| Appendix Tables |
| Table S1 Characteristics of selected GC cell lines. |
| Table S2 Clinical information of GC enrolled in this study according to CD34 expression. |
| Table S3 Statistical models of CD34 level in the YGC cohort (N=357). |
| Table S4 Subgroup analysis of YGC cohort by the tissue level of CD34. |
| Table S5 Downregulated genes in the SNU484 CD34 KD. |

# Appendix Figures

## Fig. S1 Three vessel markers of GCCs.


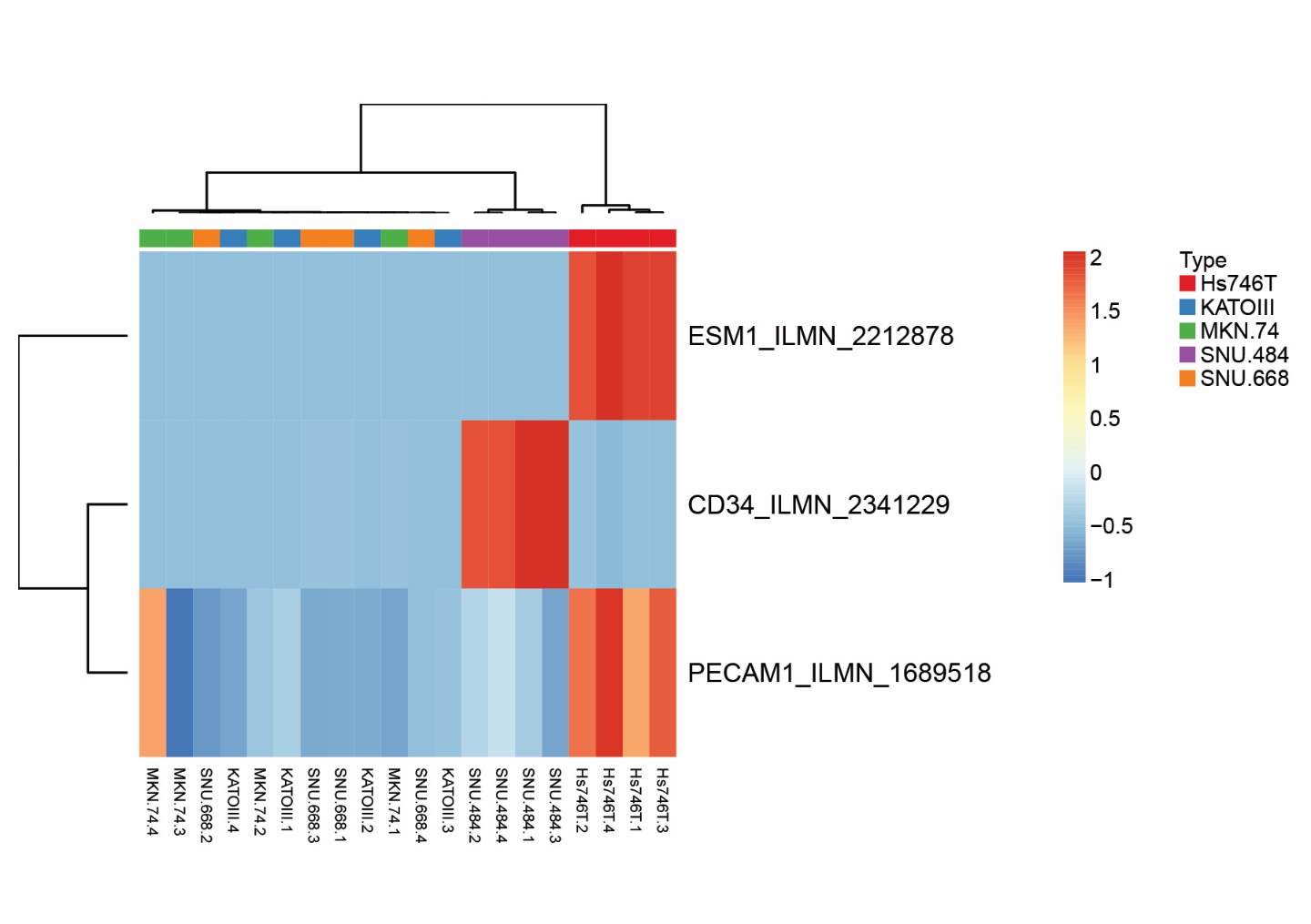


**ESM1:** Endothelial Cell Specific Molecule 1, **PECAM1:** Platelet endothelial cell adhesion molecule (or CD31). Five types of GCCs were selected from the various GCCs. The quantile normalized microarray data was scaled by row.

## Fig. S2 Correlation of ESM1 and PECAM1 in the GCCs.


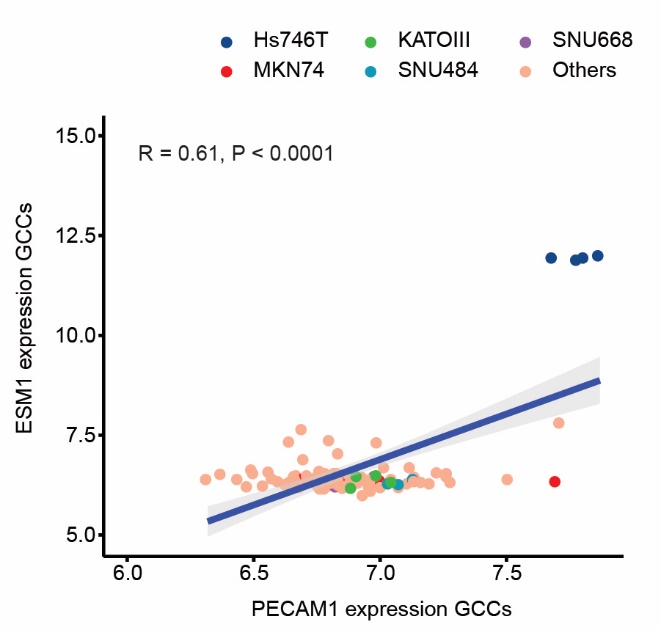


GCCs: Gastric cancer cells. Quantile normalized microarray data with selected cells marked with colors. ESM1: Endothelial Cell Specific Molecule 1, PECAM1: Platelet endothelial cell adhesion molecule (or CD31).

## Fig. S3 CD34 expression with antibody from SNU484 GCC.


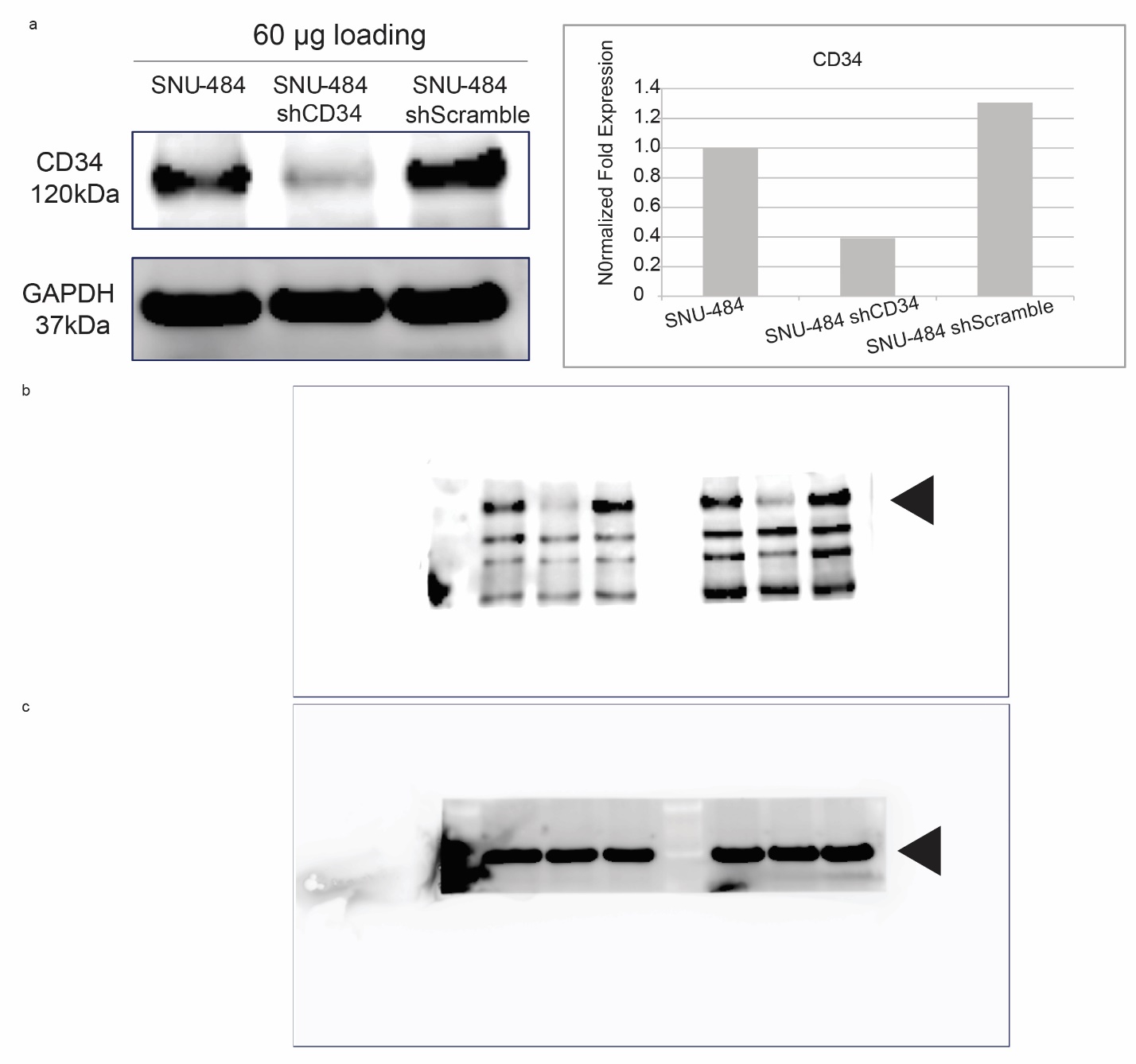


CD34(ab81289, monoclonal Ab), GAPDH (sc-47724, polyclonal Ab). a. Quantification of CD34 and GAPDH.

b. Uncropped gel of CD34. c. Uncropped gel of GAPDH. Black arrowhead indicates the cropped area.

## Fig. S4 Flow cytometry results of SNU484.


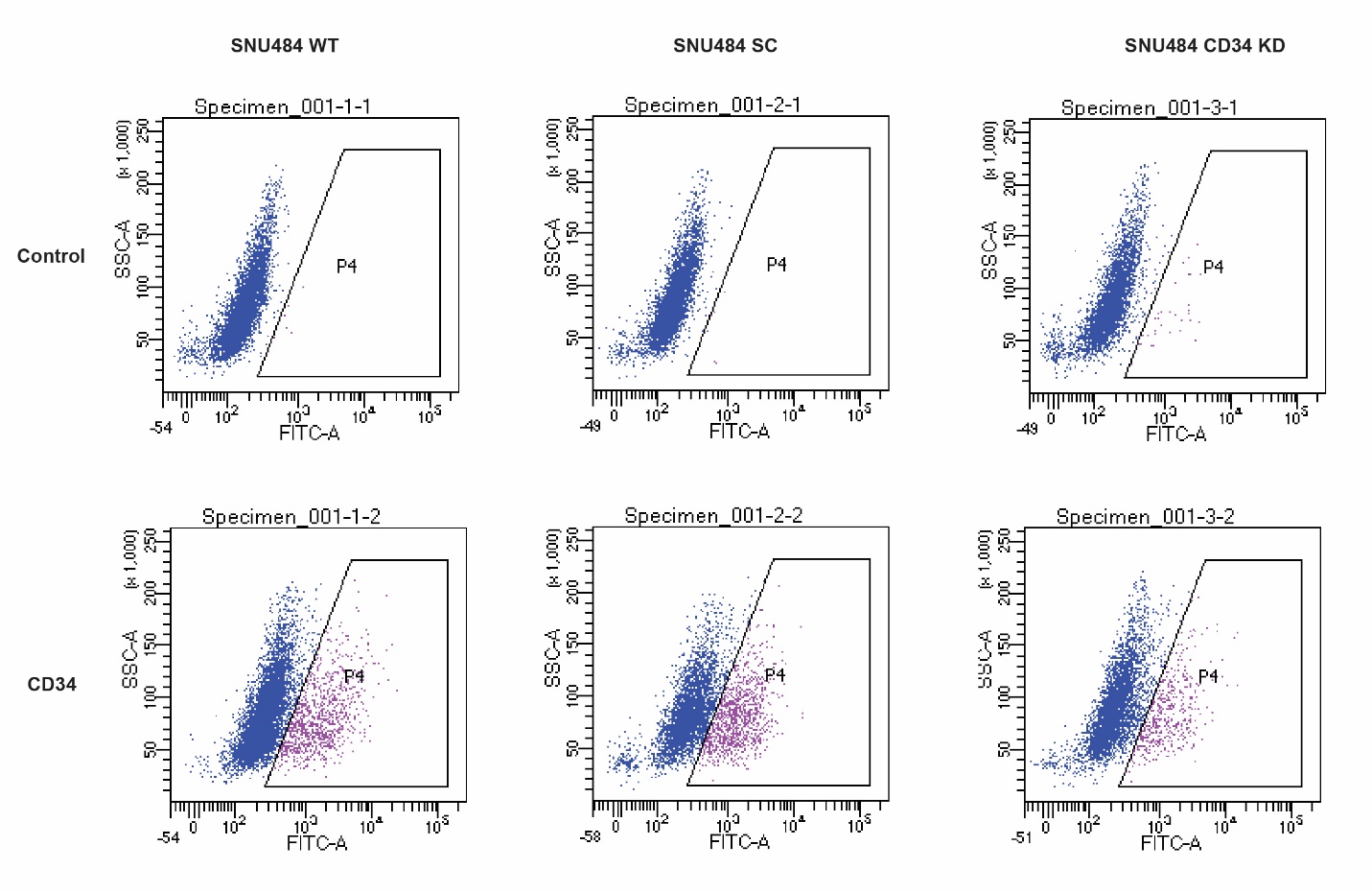


|  | SNU484 WT | SNU484 SC | SNU484 CD34 KD |
| --- | --- | --- | --- |
| Control | 0.1 | 0.1 | 0.4 |
| Ab-CD34 | 9.2 | 10.1 | 4.4 |

Ab: Antibody, WT: SNU484 wildtype, SC: Scramble shRNA, KD: Knock-down

## Fig. S5 Tumorsphere formation in the SNU484 GCCs.


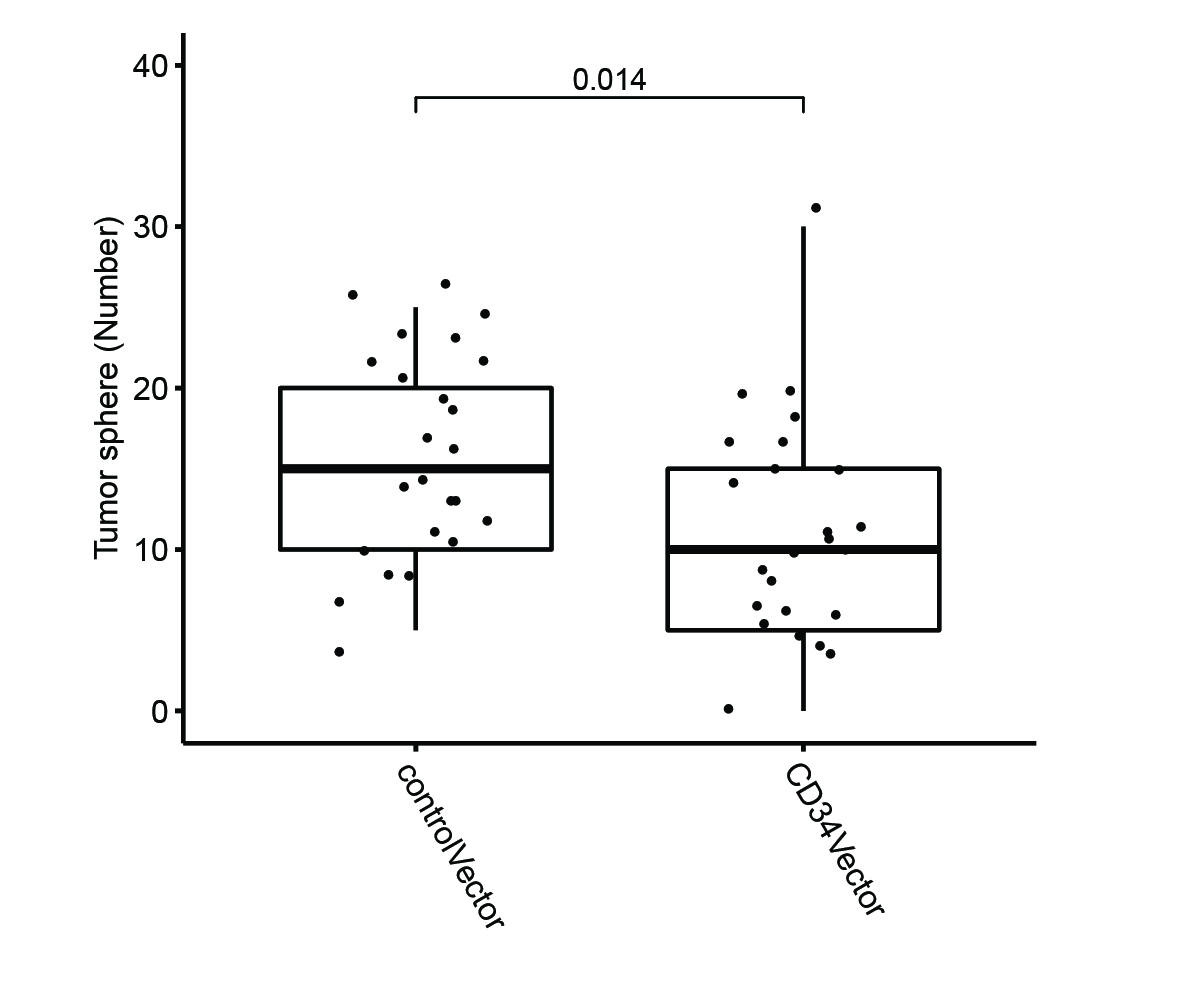


Tumorspheres were cultured in 50 μL DMEM/F12 (Gibco) supplemented with bFGF, EGF, B27, 10% FBS, and 1% antibiotics in the 96-well plate. The number of spheres were counted 30 days after the incubation.

## Fig. S6 Migration assay in the SNU484 GCCs.


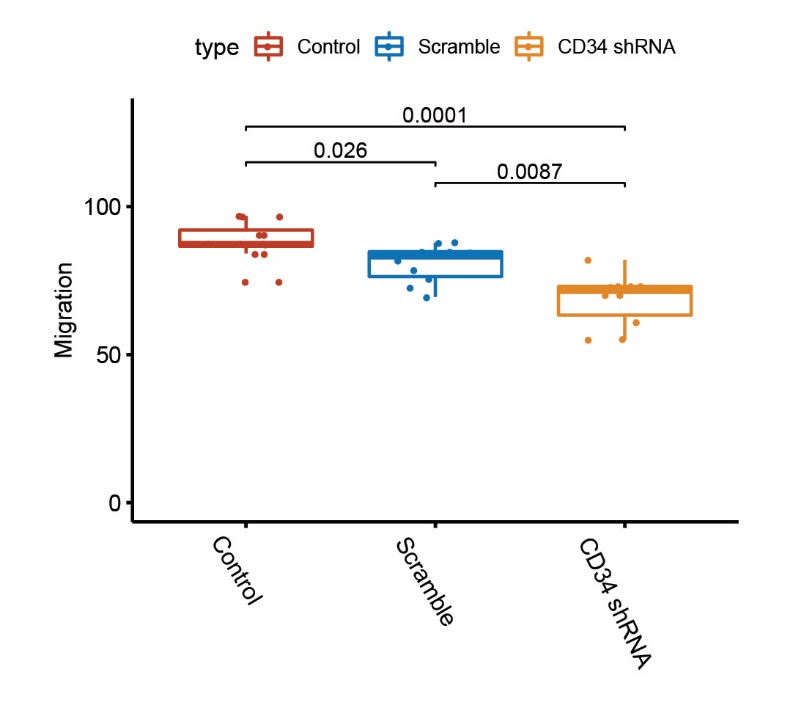


The 72h recovered wound area were normalized by the area immediately after the assay.

## Fig. S7 Individual comparison of MR images of control and CD34 KD mice.


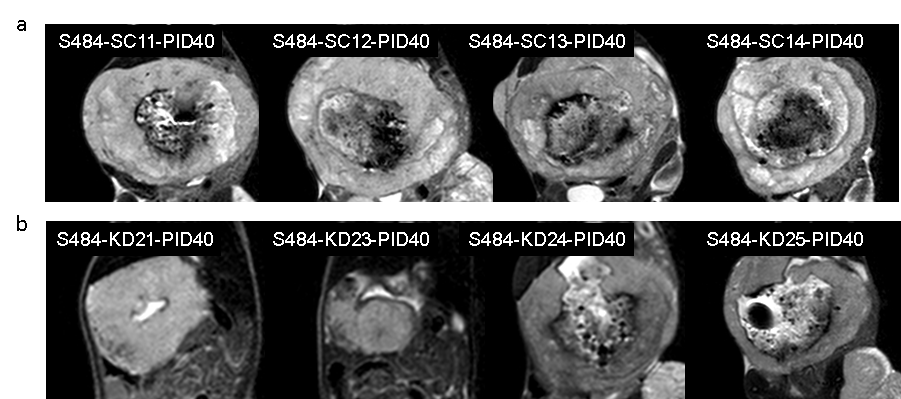


All data represents the 40 days after the injection of tumor cells. a. demonstration of four individual PID 40 SNU484 mice with scramble shRNA. b. individual mice with CD34 KD.

## Fig. S8 The tumor volume in the heterotopic models.


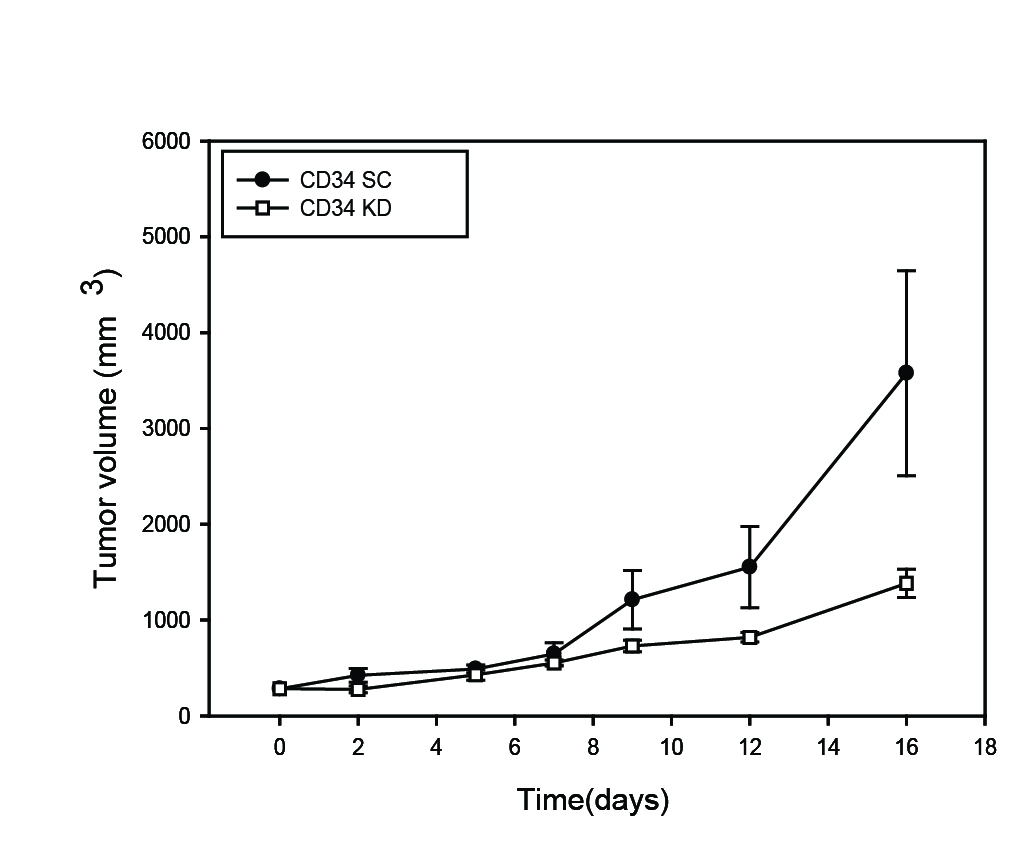


SC: shRNA scramble, KD: CD34 shRNA knock-down

## Fig. S9 The KM curve with the survival of orthotopic models.


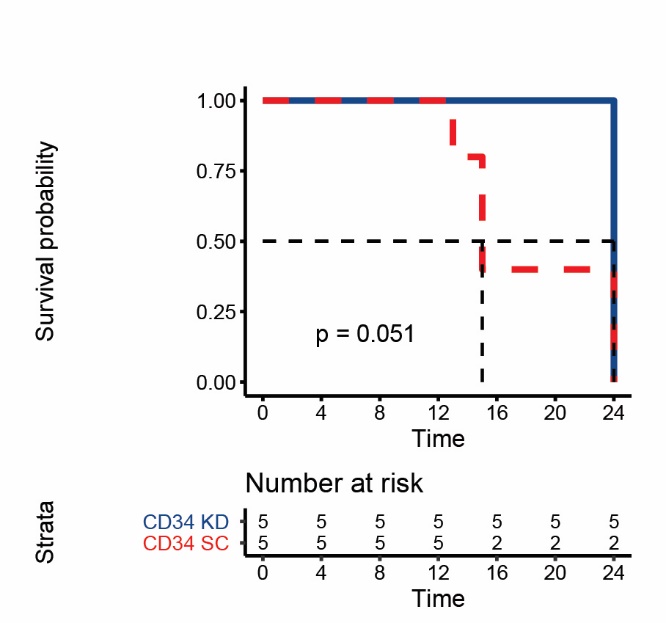


Survival was estimated by the date of four-fold enlargement of tumor from the seventh day tumor volume measurement.

## Fig. S10. Drug treatment on the SNU484.


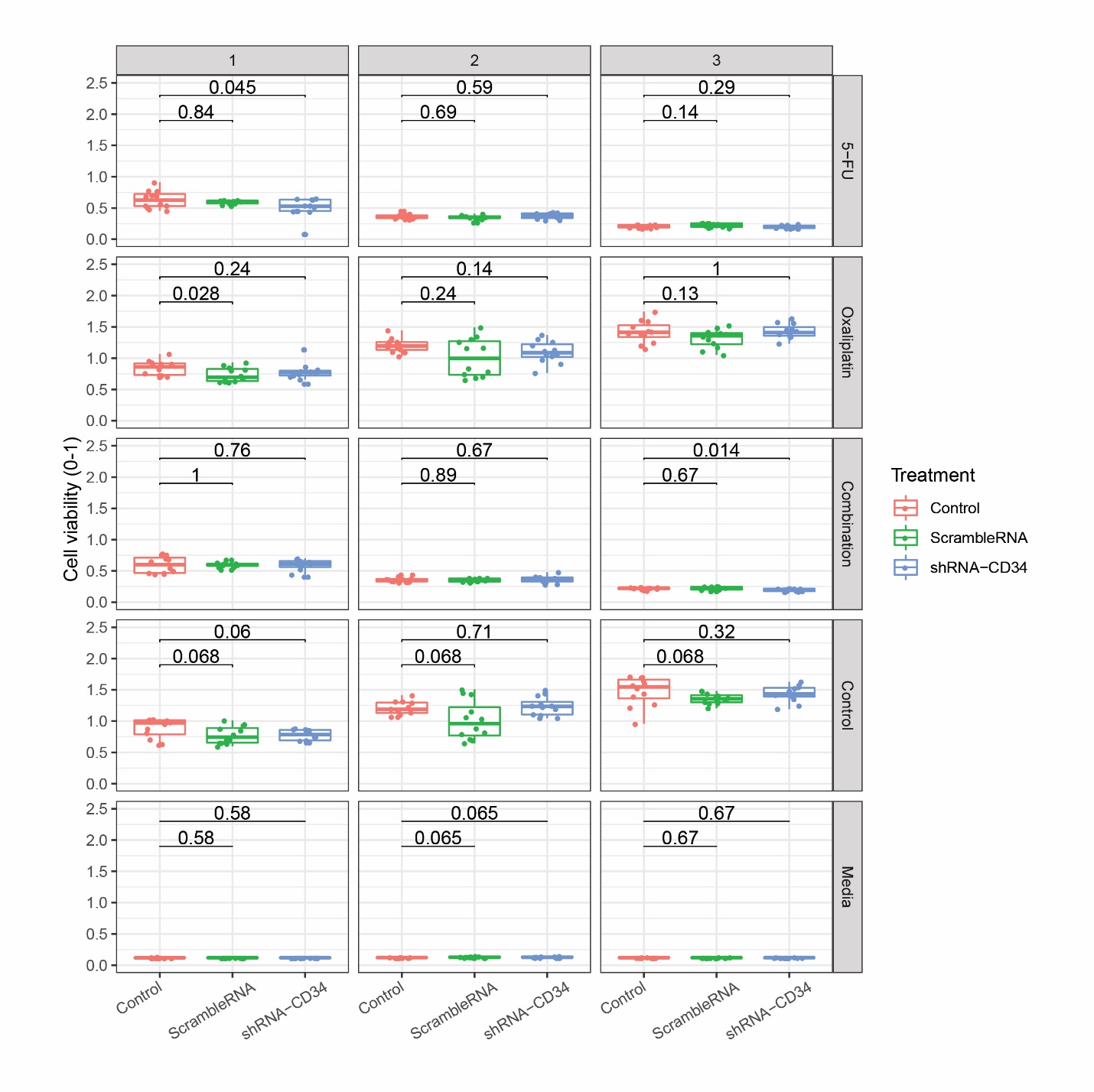


From day 1 to 3 the viability is summarized without normalization. T-test for the statistics. 5-FU: 5-Fluorourasil

## Fig. S11. RNAseq validation of the SNU484 and Hs746T.


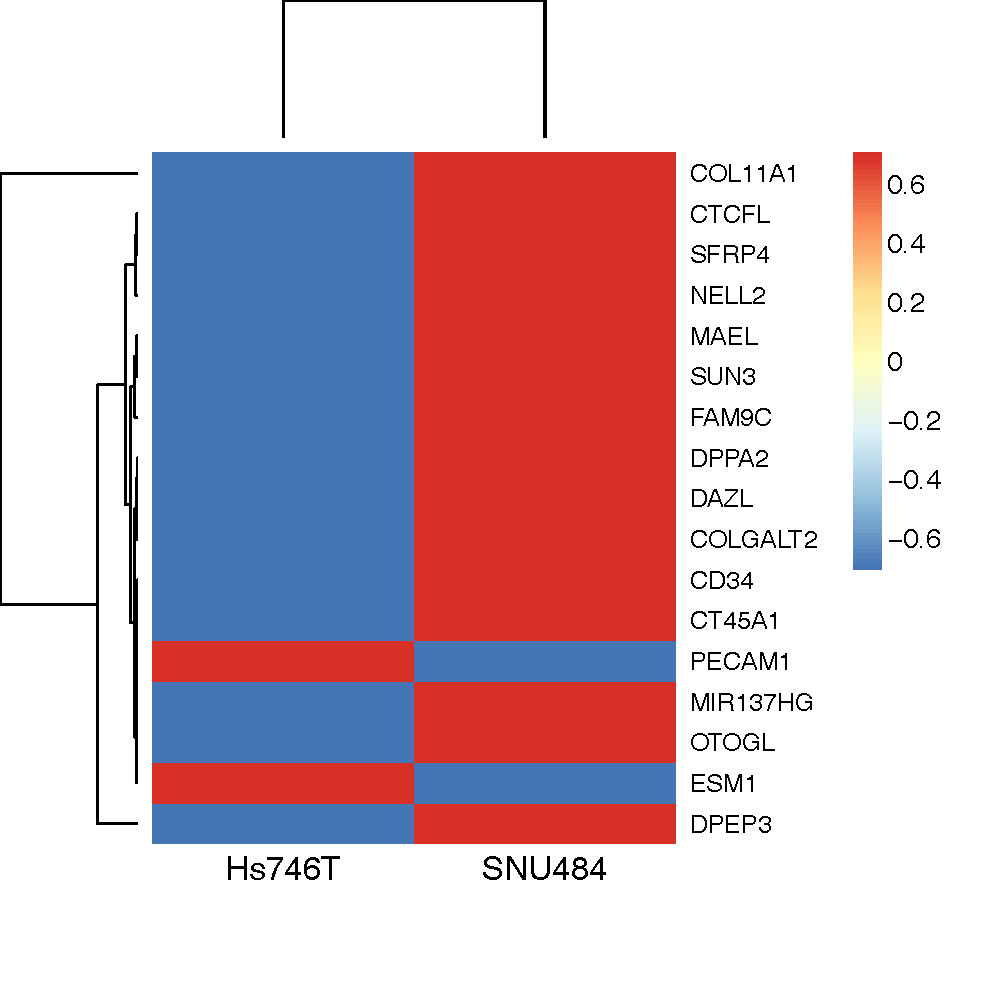


Microarray data was replicated with RNAseq. The selected genes are the SNU484-specific genes with PECAM1 and ESM1 are for Hs746T.

# Appendix Tables

## Table S1 Characteristics of selected GC cell lines.

| Group | Cell Names | Orthotopic  WT GCCs | Orthotopic  CD34 KD | Heterotopic tumor | Culture media | Growth Type |
| --- | --- | --- | --- | --- | --- | --- |
| Diffuse | SNU484 | 100 % (8/8) | 66.7 % (6/9) | 100 % (10/10) | RPMI | Adherent |
|  | SNU668 | 77.7 % (7/9) | - | 100 % (9/9) | RPMI | Adherent |
|  | Hs746T | 50% (5/10) | - | 100 % (10/10) | DMEM | Adherent |
|  | Kato III | 0 % (0/10) | - | 90 % (9/10) | RPMI | Half adherent |
| Intestinal | MKN74 | 71.4 % (5/7) | - | 100 % (7/7) | RPMI | Adherent |

Data in percent with the total number of mice with the established tumor. RPMI: RPMI 1640 media, DMEM: Dulbecco's Modified Eagle Medium. Heterotopic tumorigenicity was evaluated in the second week after injection with MRI and manual examination. Orthotopic tumors were evaluated in the four to five weeks after the injection with MRI.

## Table S2 Clinical information of GC enrolled in this study according to *CD34* expression.

| Characteristics | Entire Cohort | 1Q (The lowest) | 2Q | 3Q | 4Q (The highest) |
| --- | --- | --- | --- | --- | --- |
| Number of Patients | 357 | 90 | 89 | 89 | 89 |
| Mortality Events n (%) | 174 (48.73) | 34 (37.78) | 42 (47.19) | 44 (49.43) | 54 (60.67) |
| Age - yr | 59.51±11.31 | 60.11 ± 8.73 | 61.47 ±11.58 | 59.45±12.97 | 57.02±11.29 |
| Sex Male - % |  |  |  |  |  |
| BMI | 21.85 ± 4.33 | 22.29 ± 4.19 | 20.88 ± 4.75 | 22.63± 4.44 | 21.85±3.84 |
| Lauren % |  |  |  |  |  |
| Diffuse | 28.29 | 18.89 | 24.72 | 29.21 | 40.45 |
| Intestinal | 33.33 | 38.89 | 37.08 | 33.71 | 23.60 |
| Mixed | 3.08 | 5.56 | 4.49 | 2.25 | 0.00 |
| Other | 35.29 | 36.67 | 33.71 | 34.83 | 35.96 |
| AJCC 8^th^ edition |  |  |  |  |  |
| I A | 1.96 | 2.22 | 2.25 | 3.37 | 0.00 |
| I B | 3.36 | 4.44 | 1.12 | 3.37 | 4.49 |
| II A | 8.40 | 12.22 | 7.87 | 8.99 | 4.49 |
| II B | 17.65 | 18.89 | 23.60 | 13.48 | 14.61 |
| III A | 31.37 | 30.00 | 30.34 | 32.58 | 32.58 |
| III B | 24.37 | 23.33 | 23.60 | 22.47 | 28.09 |
| III C | 7.84 | 4.44 | 8.99 | 7.87 | 10.11 |
| IV | 5.04 | 4.44 | 2.25 | 7.87 | 5.62 |
| Histology |  |  |  |  |  |
| MD | 30.81 | 38.89 | 39.33 | 26.97 | 17.98 |
| PD | 48.18 | 36.67 | 42.70 | 51.69 | 61.80 |
| SRC | 6.72 | 6.67 | 3.37 | 5.62 | 11.24 |
| WD | 3.64 | 6.67 | 3.37 | 3.37 | 1.12 |
| Other | 10.64 | 11.11 | 11.24 | 12.36 | 7.87 |
| Tumor size (cm^3^) | 65.99 ± 26.75 | 66.16 ± 24.96 | 63.87 ± 21.27 | 68.39 ± 31.28 | 65.51 ± 28.69 |
| CCI (Overall Period, including GC) - % | | | | | |
| 1-2 | 43.14 | 50.00 | 41.57 | 42.70 | 38.20 |
| 3-4 | 15.69 | 15.56 | 13.48 | 15.73 | 17.98 |
| 5≤ | 22.97 | 17.78 | 28.09 | 21.35 | 24.72 |

The data displayed with mean plus/minus standard deviation. GC: Gastric cancer, 1Q to 4Q: First to fourth quantile, BMI: Body mass index, AJCC: Cancer classification by American Joint Committee on Cancer, MD: Moderately differentiated histology, PD: Poorly differentiated histology, SRC: Signet-ring cell histology, WD: Well-differentiated histology, CCI: Charlson comorbidity score.

## Table S3 Statistical models of *CD34* level in the YGC cohort (N=357).

| Models | Modeling variables | HR (CI 95%) | P |
| --- | --- | --- | --- |
| Model 1 | CD34 | 1.136 (0.983–1.312) | 0.082 |
| Model 2 | CD34 + Age | 1.171 (1.007–1.361) | 0.039^*^ |
| Model 3 | CD34 + Age + Gender + TNM 8^th^ | 1.205 (1.028–1.411) | 0.020^*^ |
| Model 4 | Model 3 + Tumor size + BMI | 1.241 (1.054–1.461) | 0.009^*^ |
| Model 5 | Model 4 + Lauren classification | 1.237 (1.048–1.462) | 0.012^*^ |
| Model 6 | Model 4 + Histology | 1.218 (1.026–1.446) | 0.023^*^ |
| Model 7 | Model 4 + Lauren classification + Histology | 1.218 (1.025–1.447) | 0.024^*^ |

CD34 gene expression level of tissue microarray was log2 transformed. Age and tumor size are included as the quantile. TNM 8^th^: Cancer classification by American Joint Committee on Cancer. * Statistically significant models are remarked with asterisk if P value less than 0.05.

## Table S4 Subgroup analysis of YGC cohort by the tissue level of CD34.

| Variables | Range | N | HR (95% CI) | P value |
| --- | --- | --- | --- | --- |
| Age | 27-61 | 180 | 1.037 (0.8 - 1.343) | 0.785 |
|  | 62-86 | 177 | **1.456 (1.129 - 1.878)** | **0.004** |
| Sex | Female | 115 | 1.387 (0.934 - 2.059) | 0.105 |
|  | Male | 242 | 1.181 (0.963 - 1.448) | 0.11 |
| Histology | MD | 110 | 1.167 (0.813 - 1.674) | 0.402 |
|  | Other | 75 | 0.971 (0.659 - 1.43) | 0.881 |
|  | PD | 172 | **1.502 (1.146 - 1.968)** | **0.003** |
| TNM 8^th^ | I to IIB | 112 | 1.122 (0.762 - 1.65) | 0.56 |
|  | IIIA | 112 | 1.266 (0.902 - 1.777) | 0.173 |
|  | IIIB to IV | 133 | **1.324 (1.01 - 1.734)** | **0.042** |
| Chemotherapy | Yes | 294 | 1.184 (0.969 - 1.447) | 0.098 |
|  | Other | 63 | 1.479 (0.973 - 2.247) | 0.067 |

YGC cohort: Yonsei gastric cancer cohort, TNM 8^th^: Cancer classification by American Joint Committee on Cancer, MD: Moderately differentiated histology, PD: Poorly differentiated histology, Other histology includes: Signet-ring cell histology and well-differentiated histology,

## Table S5 Downregulated genes in the SNU484 CD34 KD.

| Pathway | Total | Expected | Hits | P.Value | FDR | Genes |
| --- | --- | --- | --- | --- | --- | --- |
| Lysosphingolipid and LPA receptors | 8 | 0.0419 | 3 | 7.26E-06 | 0.0102 | S1PR1  LPAR2  S1PR5 |
| Extracellular matrix organization | 157 | 0.823 | 6 | 0.000142 | 0.0994 | COL4A5  MMP1  MMP10  PLOD2  TIMP1  COL21A1 |
| Activation of Matrix Metalloproteinases | 31 | 0.162 | 3 | 0.000536 | 0.229 | MMP1  MMP10  TIMP1 |
| Degradation of the extracellular matrix | 77 | 0.404 | 4 | 0.000653 | 0.229 | COL4A5  MMP1  MMP10  TIMP1 |
| Synthesis of PIPs at the early endosome membrane | 13 | 0.0681 | 2 | 0.00201 | 0.563 | MTM1  INPP4B |
| Degradation of collagen | 61 | 0.32 | 3 | 0.00386 | 0.809 | COL4A5  MMP1  MMP10 |
| Collagen biosynthesis and modifying enzymes | 62 | 0.325 | 3 | 0.00404 | 0.809 | COL4A5  PLOD2  COL21A1 |
| Collagen formation | 85 | 0.446 | 3 | 0.00972 | 1 | COL4A5  PLOD2  COL21A1 |
| Synthesis of PIPs at the plasma membrane | 33 | 0.173 | 2 | 0.0127 | 1 | MTM1  INPP4B |

This table includes the differentially downregulated genes in the SNU484 CD34 KD than SNU484 SC.
